# Predictive Nomogram for Clinical Prognosis in Cervical Spondylotic Myelopathy With Intramedullary T2-Weighted Increased Signal Intensity: A Novel Digital Tool for Patient Prognosis Education

**DOI:** 10.3389/fpubh.2022.898242

**Published:** 2022-05-31

**Authors:** Jie Wang, Haopeng Li, Baohui Yang

**Affiliations:** Department of Orthopedic Surgery, Second Affiliated Hospital of Xi'an Jiaotong University, Xi'an, China

**Keywords:** cervical spondylotic myelopathy, intramedullary increased signal intensity, nomogram, digital tool, patient prognosis education

## Abstract

**Aims:**

To establish a predictive nomogram for clinical prognosis in cervical spondylotic myelopathy (CSM) with intramedullary T2-weighted increased signal intensity (ISI).

**Methods:**

The clinical data of 680 patients with CSM with intramedullary T2-weighted ISI were retrospectively analyzed. The patients were divided into the modeling group (476) and the validation group (204) by using a random number table at a ratio of 7:3. The independent prognostic factors were screened using multivariate logistic regression analysis. The factors were subsequently incorporated into the establishment of the predictive nomogram. The area under the receiver operating characteristic (ROC) curve (AUC) was undertaken to estimate the discrimination of the predictive nomogram. The calibration curve and the Hosmer-Lemeshow test were used to assess the calibration of the predictive nomogram. The clinical usefulness of the predictive nomogram was evaluated by decision curve analysis (DCA).

**Results:**

Based on the pre-operative Japanese Orthopedic Association (JOA) score, maximal canal compromise (MCC), and maximal spinal cord compression (MSCC), we established a predictive nomogram. The AUCs in the modeling group and validation group were 0.892 (95% CI: 0.861~0.924) and 0.885 (95% CI: 0.835~0.936), respectively, suggesting good discrimination of the nomogram. Calibration curves showed a favorable consistency between the predicted probability and the actual probability. In addition, the values of *P* of the Hosmer-Lemeshow were 0.253 and 0.184, respectively, suggesting good calibration of the nomogram. DCA demonstrated that the nomogram had good clinical usefulness.

**Conclusion:**

We established and validated a predictive nomogram for the clinical prognosis in CSM with intramedullary T2-weighted ISI. This predictive nomogram could help clinicians and patients identify high-risk patients and educate them about prognosis, thereby improving the prognosis of high-risk patients.

## Introduction

Cervical spondylotic myelopathy (CSM) is a progressive degenerative disease defined by spondyloarthritis, congenital cervical canal stenosis, and ossification of the posterior longitudinal ligament (1–3). In the early stages of the disease, patients with CSM present mild symptoms that are easily ignored. That often causes a delayed diagnosis and irreversible neurologic damage. As the disease progresses, patients with CSM often exhibit a wide variety of symptoms and signs, such as numb hands, bilateral arm paresthesia, gait abnormality, and positive Hoffmann signs (4, 5). At present, the diagnosis of CSM is mainly based on clinical symptoms, signs, and clinical imaging. Magnetic resonance imaging (MRI) is an invaluable and irreplaceable modality for radiographic neurological assessment by visualizing the degree of spinal cord compression and the signal changes of the spinal cord (6, 7).

The presence of intramedullary increased signal intensity (ISI) was found in patients with cervical spondylotic myelopathy on T2-weighted images, suggesting spinal cord injury (8). ISI on T2-weighted MRI reflects chronic intramedullary compression lesions, such as neuronal cell death, demyelination, and reactive astrogliosis (9–11). MRI findings include intramedullary abnormal ISI on T2-weighted images indicating poor prognosis in CSM (12, 13).

At present, no consensus is reached on the independent prognostic factors of patients with CSM with intramedullary T2-weighted ISI, and it is still controversial. As a statistical predictive model, a nomogram estimates individualized risk based on independent prognostic factors. The nomogram could help clinicians and patients predict disease prognosis and timely identify the high-risk patients. Hence, we established and validated a predictive nomogram for the clinical prognosis in CSM with intramedullary T2-weighted ISI. We hope that this predictive nomogram could help clinicians and patients identify high-risk patients and educate them about prognosis, thereby improving the prognosis of high-risk patients.

## Patients and Methods

### Patients

**Figure 1:**
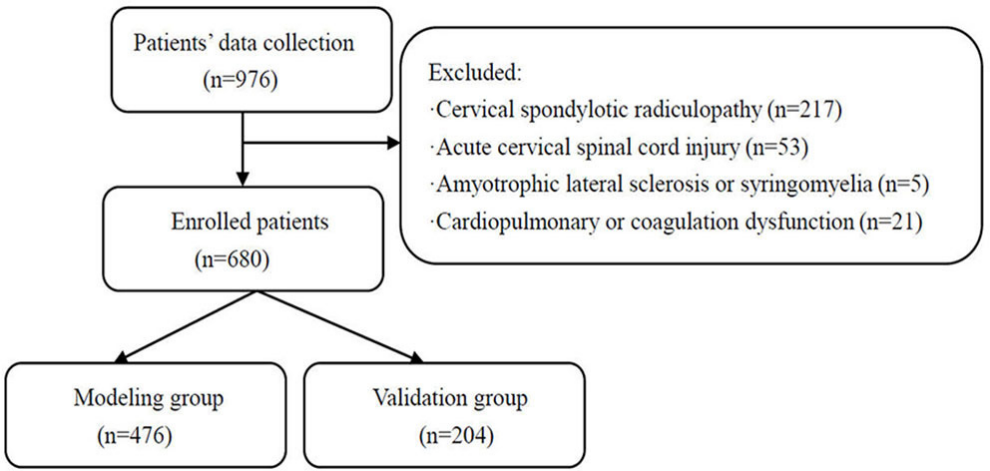
Flowchart of patients included in this study.

We retrospectively collected and analyzed the data of patients with CSM with intramedullary T2-weighted ISI admitted to the Second Affiliated Hospital of Xi'an Jiaotong University between January 2012 and June 2021. All patients underwent a detailed examination on admission, while their imaging included cervical spine plain X-rays and MRI. Patients were selected according to the following inclusion criteria: (1) aged 18 years and above, (2) patients with CSM, (3) cervical MRI indicated spinal cord compression, and (4) cervical MRI showed intramedullary T2-weighted ISI. Patients were excluded based on the following criteria: (1) cervical spondylotic radiculopathy, (2) acute cervical spinal cord injury, (3) amyotrophic lateral sclerosis or syringomyelia, and (4) cardiopulmonary or coagulation dysfunction. Finally, 680 patients were included in the final analysis. The patients who met the inclusion criteria were divided into the modeling group (476) and the validation group (204) by using a random number table at a ratio of 7:3. A flow diagram is shown in Figure 1. The modeling group was further divided into two groups based on whether the 6-month post-operative improvement rate for the Japanese Orthopedic Association (JOA) score was ≥60%, namely, the poor prognosis group (115) and the good prognosis group (361). This study was approved by the Medical Ethics Committee of the Second Affiliated Hospital of Xi'an Jiaotong University, China (approval No. 2021226). Verbal informed consent was obtained from each participant. Since the data were anonymous and the study was retrospective, no written informed consent was obtained.

### Assessment of Neurological Function

The pre-operative and post-operative neurological functions were assessed by using the JOA score. Prognosis status was evaluated by a 6-month post-operative improvement rate for the JOA score. The calculation formula of the JOA improvement rate is as follows: the JOA improvement rate = (post-operative JOA score—pre-operative JOA score)/(17—pre-operative JOA score) × 100% (14). A JOA score improvement rate of more than 75% is excellent, 50–74% is good, 25–49% is acceptable, and <25% is poor (15).

### Radiographic Assessment

**Figure 2:**
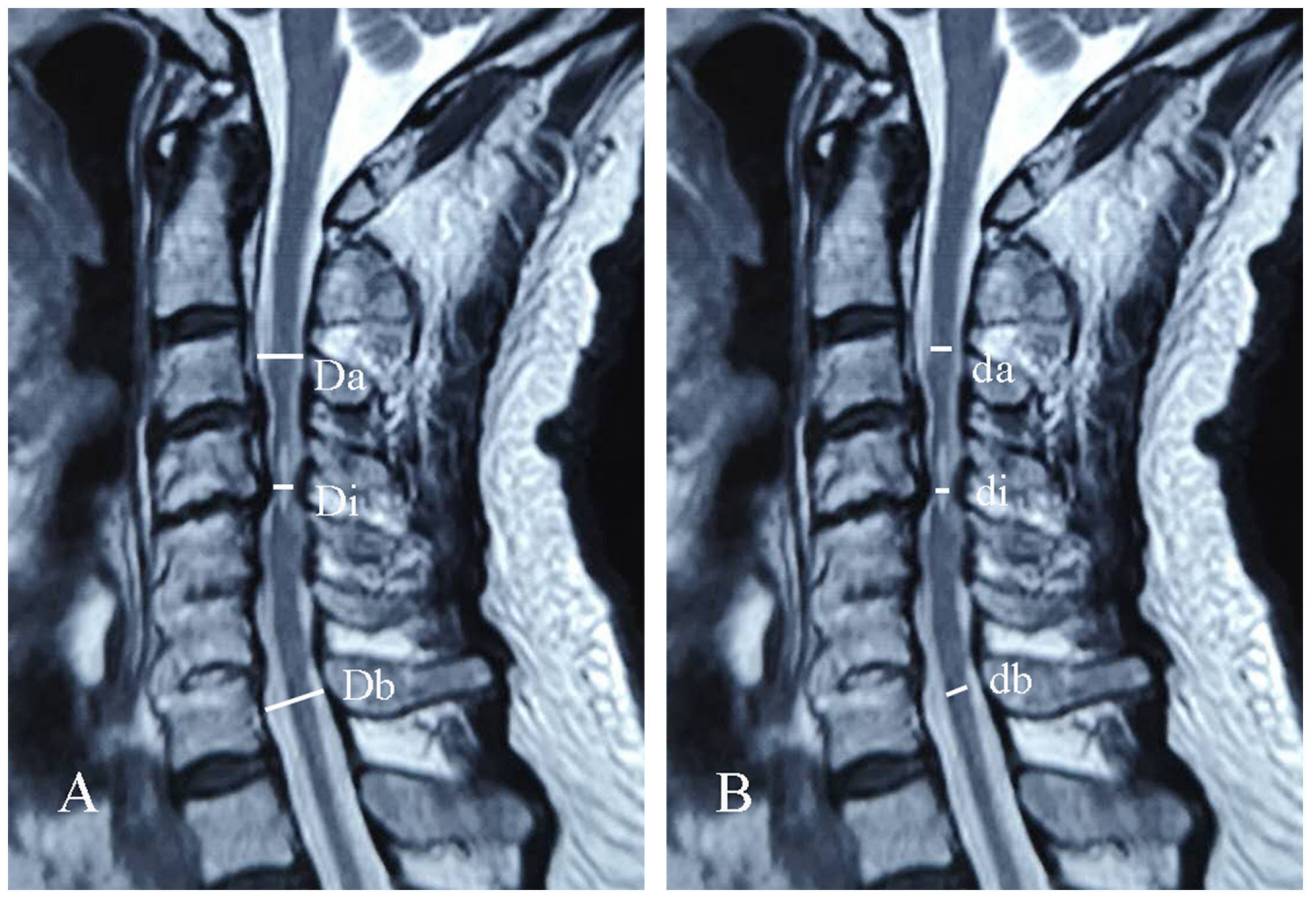
Schematic of maximal canal compromise (MCC) **(A)** and maximal spinal cord compression (MSCC) **(B)** in sagittal cervical MRI.

All patients underwent MRI on admission using either a 3.0-T MRI. Maximal canal compromise (MCC) was applied to assess the maximal canal stenosis, while maximal spinal cord compression (MSCC) was used to measure the maximal degree of spinal cord compression (16). The MCC was defined as the sagittal anteroposterior spinal canal diameter at the maximal spinal canal stenosis (Di) divided by the averaged sagittal anteroposterior spinal canal diameter between the non-pathological spinal canal above (Da) and non-pathological spinal canal below (Db) (Figure 2A). MCC was calculated as follows: MCC (%) = [1 – Di/(Da + Db)/2] × 100%. MSCC was defined as the sagittal anteroposterior spinal cord diameter at the maximal spinal cord compression (di) divided by the averaged sagittal anteroposterior spinal cord diameter between the non-pathological spinal cord above (da) and non-pathological spinal cord below (db) (Figure 2B). MSCC was calculated as follows: MSCC (%) = [1 – di/(da + db)/2] × 100%. During the radiographic assessment, radiographic measurements were obtained by two experienced researchers measuring MRI images of each patient, respectively. Student's *t*-test analysis was performed on the two groups of measurements. If there was no statistical significance, the mean values of the two groups of measurements were taken as the final radiographic measurements. Otherwise, the above assessment was repeated.

### Clinical Treatment

All patients underwent anterior or posterior cervical decompression surgery. Of these, 139 patients underwent anterior cervical corpectomy and fusion, and 541 patients underwent posterior cervical expansive open-door laminoplasty. All surgical procedures were performed by the corresponding author. The external head and neck stabilization was continued for 3 months after surgery. Patients underwent clinical review and evaluation of a neurological function at 6 months post-operatively.

### Observed Indicators

The following patients' data were collected and analyzed: gender, age, body mass index (BMI), disease duration, hypertension, diabetes, pre-operative JOA score, MCC, MSCC, number of spinal cord compression segments, operative time, intraoperative blood loss, 6-month post-operative JOA score, and 6-month post-operative improvement rate for the JOA score.

### Statistical Analysis

Statistical analyses were performed using IBM SPSS Statistics software version 26.0 (SPSS Inc., Chicago, IL, United States) and R software version 4.1.1. The measurement data were expressed as mean ± standard deviation (SD), and the enumeration data were expressed in percentages. Continuous variables were analyzed using Student's *t*-test or the Mann-Whitney *U*-test, and categorical variables were analyzed using the χ^2^ test or Fisher's exact test. The independent prognostic factors of patients with CSM with intramedullary T2-weighted ISI were screened using univariate and multivariate logistic regression analysis in the modeling group. The independent prognostic factors were subsequently incorporated into the establishment of the predictive nomogram. The predictive nomogram was validated internally in the modeling group and externally in the validation group. The area under the receiver operating characteristic (ROC) curve (AUC) was undertaken to estimate the discrimination of the predictive nomogram. The calibration curve and the Hosmer-Lemeshow test were used to assess the calibration of the predictive nomogram. The clinical usefulness of the predictive nomogram was evaluated by decision curve analysis (DCA). The value of *P* < 0.05 denoted a statistically significant difference.

## Results

### Patients' Characteristics

A total of 680 individuals, including 476 patients in the modeling group and 204 patients in the validation group, were included in the present study (Table 1). There were 361 patients whose 6-month post-operative improvement rate for the JOA score was ≥60% in the modeling group. The treatment success rate for patients with CSM with intramedullary T2-weighted ISI was 75.8%. No statistically significant differences in gender, age, BMI, and other clinical data were found between the modeling group and validation group (*P* > 0.05), which was comparable.

**Table 1:** Patients' characteristics in the modeling group and the validation group.

**Characteristics**	**Modeling group (476)**	**Validation group (204)**	***t*/*Z*/χ^2^**	***P*-Value**
**Gender**				
Male	312	127	0.676	0.411
Female	164	77		
Age	57.07 ± 7.13	56.65 ± 5.51	0.827	0.409
**BMI**				
<18.5 kg/m^2^	35	19	0.925	0.630
18.5–24 kg/m^2^	300	129		
>24 kg/m^2^	141	56		
**Disease duration**				
<2 years	215	95	0.113	0.737
≥2 years	261	109		
**Hypertension**				
No	294	130	0.234	0.629
Yes	182	74		
**Diabetes**				
No	302	121	1.037	0.309
Yes	174	83		
Pre-operative JOA score	9.96 ± 1.34	9.79 ± 1.43	1.503	0.133
MCC (%)	46.87 ± 6.54	46.06 ± 5.40	1.676	0.095
MSCC (%)	39.55 ± 5.40	38.86 ± 4.95	1.636	0.103
Number of spinal cord compression segments	2.99 ± 0.75	2.90 ± 0.70	1.568	0.117
**Operative time**				
<3 h	225	98	0.034	0.854
≥3 h	251	106		
**Intraoperative blood loss**				
<100 ml	257	117	0.652	0.419
≥100 ml	219	87		

*BMI, body mass index; JOA, Japanese Orthopedic Association; MCC, maximal canal compromise; MSCC, maximal spinal cord compression*.

### Univariate and Multivariate Logistic Regression Analysis

The modeling group was further divided into two groups based on whether the 6-month post-operative improvement rate for the JOA score was ≥60%, namely, the poor prognosis group (115) and the good prognosis group (361). Disease duration, pre-operative JOA score, MCC, and MSCC were statistically significant risk factors after univariate logistic regression analysis in the modeling group (Table 2). A multivariate logistic regression analysis was performed for statistically significant risk factors. Multivariate logistic regression analysis revealed that pre-operative JOA score [odds ratio (OR) = 1.601, *P* < 0.05], MCC (OR = 1.285, *P* < 0.05), and MSCC (OR = 1.611, *P* < 0.05) were independent prognostic factors (Table 3). The logistic regression model is Logit (*P*) = −33.322 + 0.470 Pre-operative JOA score + 0.251 MCC + 0.477 MSCC.

**Table 2:** Results of univariate logistic regression analysis in the modeling group.

**Characteristics**	**Poor prognosis group (115)**	**Good prognosis group (361)**	***t*/*Z*/χ^2^**	***P*-Value**
**Gender**				
Male	68	244	2.764	0.096
Female	47	117		
Age	57.23 ± 7.06	57.01 ± 7.17	0.086	0.769
**BMI**				
<18.5 kg/m^2^	9	26	0.093	0.761
18.5–24 kg/m^2^	73	227		
>24 kg/m^2^	33	108		
**Disease duration**				
<2 years	42	173	4.538	0.033^*^
≥2 years	73	188		
**Hypertension**				
No	78	216	2.359	0.125
Yes	37	145		
**Diabetes**				
No	72	230	0.046	0.831
Yes	43	131		
Pre-operative JOA score	8.80 ± 0.91	10.34 ± 1.02	1.461	<0.001^*^
MCC	48.31 ± 5.47	42.23 ± 3.88	0.255	<0.001^*^
MSCC	42.40 ± 3.42	37.30 ± 2.94	0.520	<0.001^*^
Number of spinal cord compression segments	3.23 ± 0.74	2.61 ± 0.59	1.211	0.271
**Operative time**				
<3 h	52	173	0.256	0.613
≥3 h	63	188		
**Intraoperative blood loss**				
<100 ml	57	200	1.196	0.274
≥100 ml	58	161		

* *P < 0.05. BMI, body mass index; JOA, Japanese Orthopedic Association; MCC, maximal canal compromise; MSCC, maximal spinal cord compression*.

**Table 3:** Results of multivariate logistic regression analysis in the modeling group.

**Variable**	**B**	**SE**	**Wald**	**OR**	**95% CI**	***P*-Value**
Pre-operative JOA score	0.470	0.197	5.702	1.601	1.088~2.355	0.017^*^
MCC	0.251	0.035	50.270	1.285	1.199~1.377	<0.001^*^
MSCC	0.477	0.062	59.608	1.611	1.427~1.818	<0.001^*^
Constant	−33.322	3.561	87.550	0.000	/	<0.001^*^

* *P < 0.05. JOA, Japanese Orthopedic Association; MCC, maximal canal compromise; MSCC, maximal spinal cord compression*.

### Development of the Predictive Nomogram

**Figure 3:**
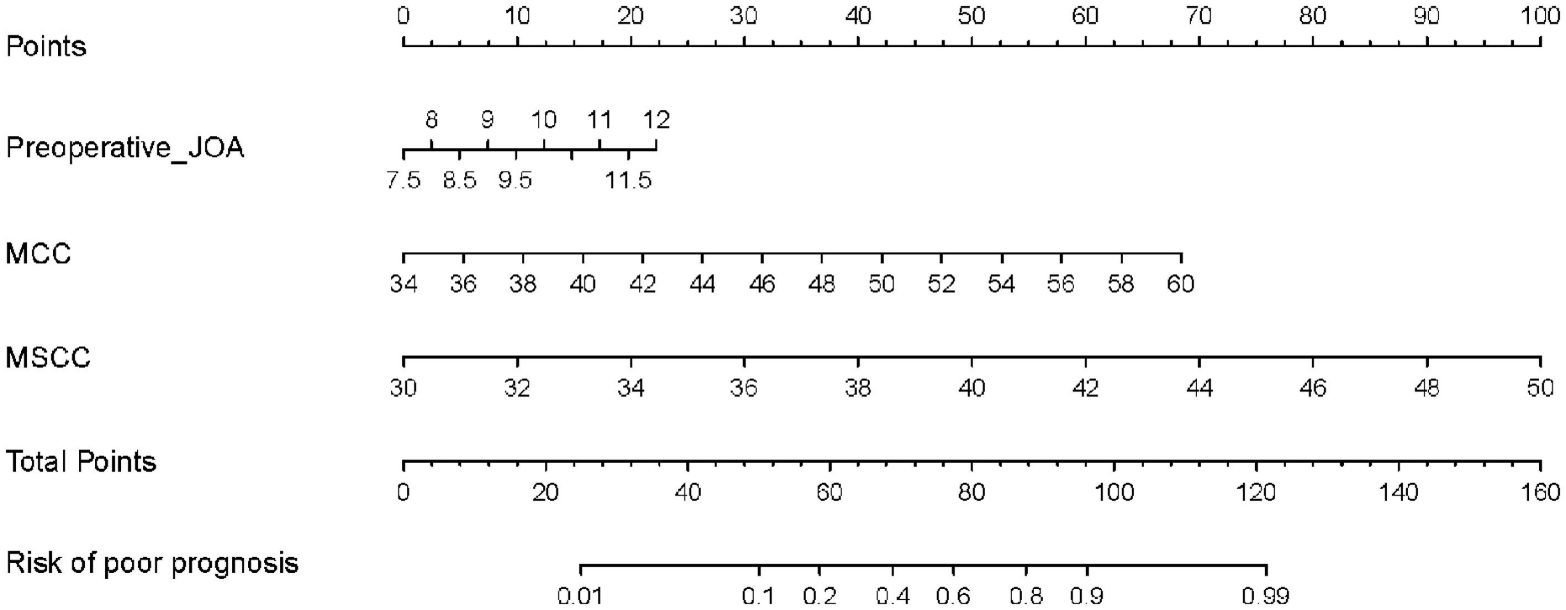
Predictive nomogram for clinical prognosis in cervical spondylotic myelopathy (CSM) with intramedullary T2-weighted increased signal intensity (ISI).

The independent prognostic factors were subsequently incorporated into the development of the predictive nomogram of patients with CSM with intramedullary T2-weighted ISI (Figure 3). In the predictive nomogram, the points corresponding to each independent prognostic factor were obtained, then the sum of the points was calculated as the total score, and the predicted risk corresponding to the total score was the probability of poor prognosis of patients with CSM with intramedullary T2-weighted ISI.

### Validation of the Predictive Nomogram

#### Discrimination

**Figure 4:**
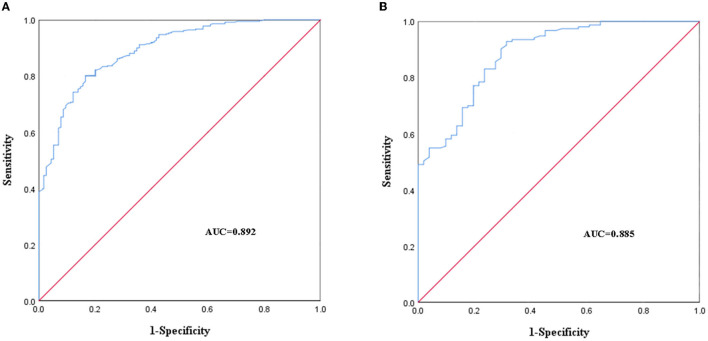
Receiver operating characteristic (ROC) curves of the predictive nomogram in the modeling group **(A)** and the validation group **(B)**.

The AUC of the modeling group was 0.892 (95% CI: 0.861~0.924), *P* < 0.001 (Figure 4A). The cutoff value of the modeling group was 0.775, *P* < 0.001. The AUC of validation group was 0.885 (95% CI: 0.835~0.936), *P* < 0.001 (Figure 4B). The results of the AUC indicated that the discrimination of the predictive nomogram was good.

#### Calibration

**Figure 5:**
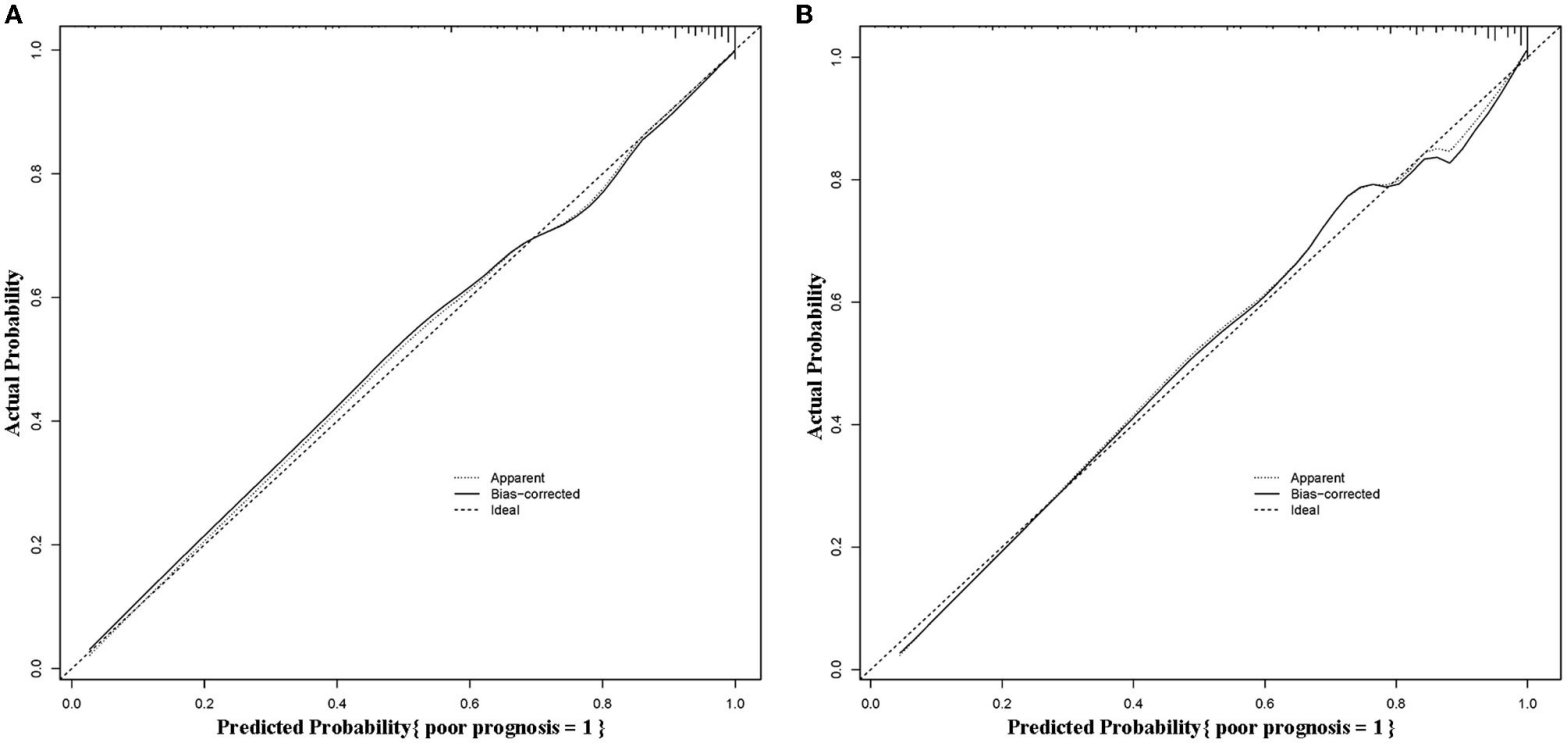
Calibration curves of the predictive nomogram in the modeling group **(A)** and the validation group **(B)**.

The calibration curves of the predictive nomogram showed a favorable consistency between the predicted probability and the actual probability in the modeling group (Figure 5A) and the validation group (Figure 5B). In addition, the results of the Hosmer-Lemeshow in the modeling group and the validation group were χ^2^ = 10.180 (*P* = 0.253) and χ^2^ = 11.319 (*P* = 0.184), respectively, suggesting the good calibration of the predictive nomogram.

#### Clinical Usefulness

**Figure 6:**
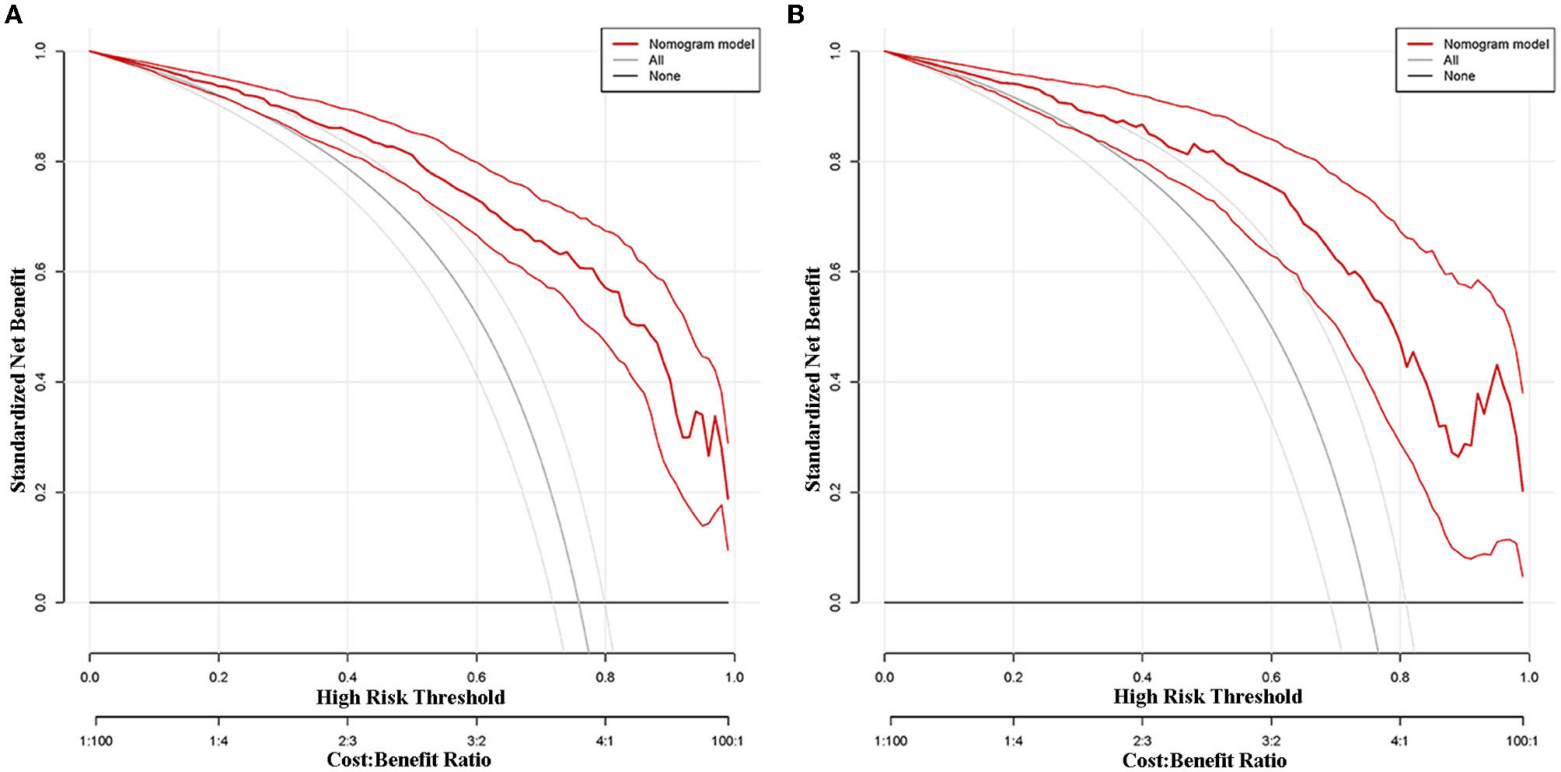
Decision curve analysis (DCA) of the predictive nomogram in the modeling group **(A)** and the validation group **(B)**.

A DCA of the predictive nomogram in the modeling group and the validation group is shown in Figure 6. DCA demonstrated that the predictive nomogram in the modeling group (Figure 6A) and the validation group (Figure 6B) presented similar net benefits at the range of threshold probability, with better net benefits than the two extreme lines when the threshold probability was 0.1~1.0. The black horizontal extreme line represented that in patients who received no intervention, the net benefit was 0. The black oblique extreme line represented that in patients who received the intervention, the net benefit was a backslash with a negative slope. The predictive nomogram had good clinical usefulness when it was in the above threshold probability. The cutoff value (0.775) obtained from the ROC curve of the modeling group was within the threshold probability range of the above two DCA curves, also indicating that the predictive nomogram has good clinical usefulness. Further analysis of the DCA curves of the predictive nomogram showed that the net clinical benefit of the modeling group and the validation group was 61 and 56%, respectively, when 0.775 was set as the threshold probability value for diagnosing poor prognosis and taking intervention. In other words, 61 and 56 of every 100 patients with CSM with intramedullary T2-weighted ISI who were diagnosed with poor prognosis using the predictive nomogram in the modeling group and the validation group would respectively have clinical benefits.

### Visualization Application of the Predictive Nomogram

**Figure 7:**
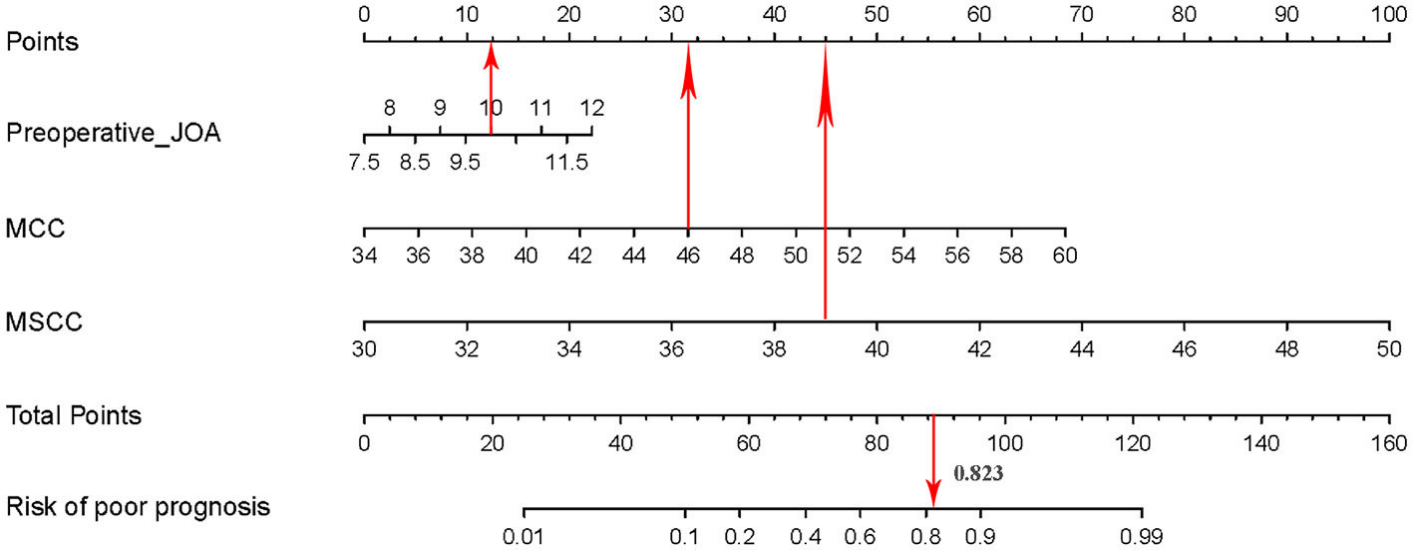
Visualization application of the predictive nomogram.

Take a patient with CSM with intramedullary T2-weighted ISI as an example. The prognostic factors for this patient were as follows: pre-operative JOA score = 10, MCC = 46, and MSCC = 39. The risk of poor prognosis for the patient was 0.823 (>0.775) according to the predictive nomogram (Figure 7). According to the DCA curve, effective interventions should be taken to reduce the risk of poor prognosis.

## Discussion

Cervical spondylotic myelopathy is a progressive, degenerative spine disease, and the most common cause of spinal cord dysfunction in adults worldwide (17). An MRI revealed cervical spinal stenosis and signal change of the cervical spinal cord. T2-weighted MRI showed corresponding intramedullary ISI suggesting cervical spinal cord injury (18, 19). From a histopathological view, the intramedullary ISI reflected the severity and extent of spinal cord lesions (20, 21). Orthopedic surgeons focused on the clinical prognostic factors of the patients with CSM with intramedullary T2-weighted ISI. The clinical prognosis of such patients was affected by various factors and was difficult to predict. In this study, we established and validated a predictive nomogram for the clinical prognosis in CSM with intramedullary T2-weighted ISI. Univariate and multivariate logistic regression analysis revealed that pre-operative JOA score, MCC, and MSCC were independent prognostic factors.

Patients with CSM who had intramedullary T2-weighted ISI presented with typical clinical symptoms, such as loss of hand dexterity, unstable gait, and sensory deficit in limbs (22, 23). A lot of quantitative measures that assess the severity of CSM have been developed to assess neurological function. Among them, the JOA score has been greatly popularized in spinal surgery (24). Zhang et al. (25) found that patients with CSM with intramedullary T2-weighted ISI usually had lower pre-operative JOA scores and less improved neurological function after surgery. The results of the present study revealed that the pre-operative JOA score was the independent prognostic factor of patients with CSM with intramedullary T2-weighted ISI. The pre-operative JOA score was incorporated into the development of the predictive nomogram of patients with CSM with intramedullary T2-weighted ISI. The predictive nomogram demonstrated a good degree of discrimination, calibration, and clinical usefulness. Patients with CSM with intramedullary T2-weighted ISI had lower pre-operative JOA scores and a higher probability of poor clinical prognosis after assessment by the predictive nomogram. The reasons causing the above phenomena might be that patients with lower pre-operative JOA scores usually had a poor pre-operative neurological function and delayed or even lost recovery of neurological function during the process of post-operative recovery. Therefore, it is necessary to predict the clinical prognosis of patients with CSM with intramedullary T2-weighted ISI using the pre-operative JOA score.

Maximal canal compromise is often used to evaluate the severity of cervical spinal stenosis in CSM. The measurement of MCC was that the anteroposterior canal diameter on midsagittal and axial T2-weighted images at the maximum compromise level was compared with the anteroposterior canal diameter at normal levels immediately above and below the level of injury (26, 27). Aarabi et al. (28) identified that there was a correlation between MCC and American Spinal Injury Association (ASIA) motor score recovery with a 1-year follow-up. In addition, they also found an inverse relationship between the degree of canal compromise and follow-up ASIA motor score. The results of the present study revealed that the MCC was the independent prognostic factor of patients with CSM with intramedullary T2-weighted ISI. The MCC was incorporated into the development of the predictive nomogram of patients with CSM with intramedullary T2-weighted ISI. After the validation of the predictive nomogram, it was found that patients with severe MCC had a greater risk of poor clinical prognosis. The reason for the above phenomena can be that the space between the spinal cord and spinal canal is severely limited when there issevere MCC. As a result, the spinal cord was more susceptible to ischemic injury resulting from compression. Furthermore, due to the persistent limitation of the spinal cord in severe MCC, the MCC was prone to progressive injury. Therefore, the post-operative recovery of neurological function in such patients was often unsatisfactory. Orthopedic surgeons should pay close attention to the severity of MCC in patients with CSM with intramedullary T2-weighted ISI in clinical diagnosis and treatment.

Maximal spinal cord compression is often used to evaluate the severity of cervical spinal cord compression in CSM. Tarawneh et al. (29) found that MSCC offered a certain prognostic value for the recovery of neurological function. Miyanji et al. (30) found that the extent of MSCC was significantly different between patients with complete and incomplete spinal cord injuries, with more substantial MSCC seen in the patients with complete spinal cord injury. The results of the present study revealed that the MSCC was the independent prognostic factor of patients with CSM with intramedullary T2-weighted ISI. The MSCC was incorporated into the development of the predictive nomogram of patients with CSM with intramedullary T2-weighted ISI. After the validation of the predictive nomogram, it was found that patients with more severe MSCC were more likely to have a poor clinical prognosis. The abovementioned phenomenon might be caused by the compression and the secondary ischemia of the spinal cord. That exacerbated the severity of spinal cord injury. Hence, the post-operative recovery of neurological function in such patients was often unsatisfactory. In clinical treatment, orthopedic surgeons should relieve or even remove spinal cord compression in a timely manner to improve the clinical prognosis of spinal cord injury.

There are some limitations to this study. First of all, this is a retrospective study. Further prospective studies are needed at a later stage. Second, this study was mainly carried out in one hospital at the present stage. A multi-center study has not been carried out. In future studies, the research team will conduct a prospective multi-center study. That will be the further improvement and verification of this study.

## Conclusion

In conclusion, we established and validated a predictive nomogram for the clinical prognosis in CSM with intramedullary T2-weighted ISI. This predictive nomogram could help clinicians and patients identify high-risk patients and educate them about prognosis, thereby improving the prognosis of high-risk patients. Patients can predict their own prognosis based on their own clinical data related to the independent prognostic factors. Then, patients can consult with their doctors as early as possible and participate in the elaboration of therapeutic protocols.

## Data Availability Statement

The raw data supporting the conclusions of this article will be made available by the authors, without undue reservation.

## Ethics Statement

The studies involving human participants were reviewed and approved by the Medical Ethics Committee of the Second Affiliated Hospital of Xi'an Jiaotong University, China. The patients/participants provided their written informed consent to participate in this study.

## Author Contributions

JW and HPL contributed to study conception and design. JW and BHY contributed to data collection, data analysis, and manuscript drafting. All authors were involved in the revision of the manuscript and approved the final version of the article.

## Funding

This study was supported by the Fundamental Research Funds for the Central Universities, No. ZRZD2017008 (to HPL).

## Conflict of Interest

The authors declare that the research was conducted in the absence of any commercial or financial relationships that could be construed as a potential conflict of interest.

## Publisher's Note

All claims expressed in this article are solely those of the authors and do not necessarily represent those of their affiliated organizations, or those of the publisher, the editors and the reviewers. Any product that may be evaluated in this article, or claim that may be made by its manufacturer, is not guaranteed or endorsed by the publisher.
